# STAT1-mediated inhibition of FOXM1 enhances gemcitabine sensitivity in pancreatic cancer

**DOI:** 10.1042/CS20180816

**Published:** 2019-03-01

**Authors:** Chao Liu, Jiaqi Shi, Qingwei Li, Zhiwei Li, Changjie Lou, Qi Zhao, Yuanyuan Zhu, Fei Zhan, Jie Lian, Bojun Wang, Xin Guan, Lin Fang, Zengxun Li, Yifei Wang, Bodong Zhou, Yuanfei Yao, Yanqiao Zhang

**Affiliations:** 1 Department of Gastrointestinal Medical Oncology, Harbin Medical University Cancer Hospital, No. 150, Haping Road, Nangang District, Harbin 150001, China; 2 Translational Medicine Research and Cooperation Center of Northern China, Heilongjiang Academy of Medical Sciences, No.194, Xuefu Road, Nangang District, Harbin 150000, China; 3 Department of Pancreatic Cancer, Tianjin Medical University Cancer Institute and Hospital, National Clinical Research Center for Cancer, Key Laboratory of Cancer Prevention and Therapy, Huan-Hu-Xi Road, Hexi District, Tianjin 300060, China

**Keywords:** FOXM1, Gemcitabine, IFNγ, Pancreatic cancer, STAT1

## Abstract

Forkhead box protein M1 (FOXM1) was identified as an oncogenic transcription factor and master regulator of tumor progression and metastasis. FOXM1 expression often correlates with poor prognosis and chemotherapy resistance. In the present study, we investigated the association of FOXM1 expression and chemoresistance in pancreatic cancer. Elevated FOXM1 protein levels were associated with gemcitabine chemoresistance in patients with pancreatic cancer. In gemcitabine resistance cell line models of pancreatic cancer, FOXM1 expression increased, which induced gemcitabine chemoresistance *in vitro*. In pancreatic cancer cells treated with gemcitabine, FOXM1 affected nuclear factor κB (NF-κB) signaling activity. Immunohistochemical analysis demonstrated a negative association of FOXM1 expression and the level of phosphorylated signal transducer and activator of transcription 1 (pSTAT1) in human pancreatic cancer tissues. Dual-luciferase reporter assays and chromatin-immunoprecipitation assays demonstrated that pSTAT1 directly binds to the *FOXM1* promoter to down-regulate its transcription. Interferon γ (IFNγ) promoted gemcitabine-induced cell apoptosis and inhibited cell proliferation *in vitro* and *in vivo* by FOXM1 inhibition. These data suggested that FOXM1 enhances chemoresistance to gemcitabine in pancreatic cancer. IFNγ could be used to down-regulate the expression of FOXM1 through STAT1 phosphorylation, thereby increasing the sensitivity of pancreatic cancer cells to gemcitabine. These studies suggested the sensitization by IFNγ in pancreatic ductal adenocarcinoma (PDAC) chemotherapy, which requires further clinical studies.

## Introduction

Pancreatic ductal adenocarcinoma (PDAC) is one of the most aggressive human cancers and has the fourth highest mortality rate amongst all tumors [1]. Although surgery and chemotherapy for PDAC have improved greatly, the 5-year overall survival (OS) rate of PDAC remains less than 7% [2]. PDAC lacks early symptoms; therefore, most patients present with metastasis, and for many cases treated with surgery, the tumor recurs within 1–2 years and develops metastasis [3]. For these patients, gemcitabine-based chemotherapy is the priority treatment; however, the cancer cells develop chemoresistance, resulting in poor efficacy of gemcitabine. This dismal prognosis indicates the urgent need for strategies to enhance chemosensitivity to gemcitabine, which would improve the outcome of chemotherapy.

Forkhead box protein M1 (FOXM1) is a member of the Forkhead box transcription factor superfamily and is a key cell-cycle regulator for both the transition from G_1_ to S phase and progression from G_2_ phase to mitosis [4]. FOXM1 expression is elevated in most human cancers and plays a crucial role in human carcinogenesis [5–7]. In addition, higher expression of FOXM1 is associated with poor prognosis of patients with cancer and can serve as an independent predictor of poor survival in cancer [8–10]. Therefore, studies have focussed on the roles of FOXM1 in regulating drug resistance in chemotherapy [11]. Research has suggested that FOXM1 can promote resistance through enhancing DNA damage repair, resistance to apoptosis, elimination of reactive oxygen species (ROS), and influencing tumor stemness [12–16]. Therefore, targetting FOXM1 would be a promising strategy to increase the chemotherapeutic effect of gemcitabine in pancreatic cancer. As a proliferation-associated transcription factor, FOXM1 plays pivotal roles in the development of PDAC via cross-talk with several critical signaling pathways [17]. However, the role of FOXM1 in advanced pancreatic cancer, especially the development of acquired chemoresistance against chemotherapeutic agents such as gemcitabine, has not yet been determined.

Previous studies have revealed that FOXM1 expression is driven primarily by the Hedgehog, Ras/MEK/MAPK, signal transducer and activator of transcription 3 (STAT3), and wnt/β-catenin in different cancer cells [18–24]. Overactivation of STAT3 is not only crucial for tumorigenesis, but also contributes to acquired resistance of tumors [25–28]. In addition, STAT1 and STAT3 also antagonize each other’s activity by competitively binding to the same target genes [29,30]. STAT3 activates FOXM1 expression, which begs the question, is there a regulatory relationship between STAT1 and FOXM1? However, current research does not suggest that STAT1 regulates FOXM1 expression.

In the present study, we found that pancreatic cancer patients with elevated FOXM1 expression has a poor prognosis and poor response to gemcitabine-based chemotherapy. FOXM1 can cause resistance to apoptosis by activating nuclear factor κB (NF-κB) pathway in pancreatic cancer cell lines. Further studies reveled that STAT1 can inhibit the expression of FOXM1 by binding to the promoter of FOXM1. Therefore, blocking STAT1/FOXM1/NFkB axis by interferon γ (IFNγ) can increase the sensitivity of pancreatic cancer to gemcitabine. A better understanding of this process might lead to the development of new methods to enhance the effect of gemcitabine treatment in this aggressive cancer.

## Materials and methods

### Patients and samples

In the present study, we randomly selected 93 patients with advanced pancreatic cancer who received gemcitabine-based monotherapy or combined chemotherapy at the Harbin Medical University Cancer Hospital, from January 2006 to December 2014 (the detailed clinicopathological features of the patients are shown in Supplementary Table S1). All the tumor samples were obtained before chemotherapy and were stored at −80°C until analysis. All patients were followed-up until January 2017, with a median observation time of 13.30 months. The present study was approved by the Ethics Committee of Harbin Medical University Cancer Hospital.

### Cell culture, establishment of gemcitabine-resistant cell line, and gene knockout

Human pancreatic cancer cell lines (SW1990, BxPC3, AsPC1, Capan1, Capan2, CFPAC1, HPAFⅡ, Hs766T, MIAPaCa2, Panc1, Panc03.27, PL45, and PSN1) and the normal pancreatic ductal epithelial cells (HPDE6.C7) were purchased from the American Type Culture Collection in 2016. All cell lines have been authenticated yearly, and recently in March 2018. These cell lines of SW1990, BxPC3, Panc1, PL45, and Hs766T were cultured in Dulbecco’s modified Eagle’s medium (DMEM, Gibco, Carlsbad, CA, U.S.A.) with 10% FBS (Gibco). MiaPaCa-2 cells were cultured in DMEM supplemented with 10% FBS and 2.5% horse serum. CFPAC1 and Capan1 cells were maintained in IMDM (Gibco) supplemented with 10% FBS. Capan2 was cultured in McCoy’s 5A medium (Gibco). The medium was supplemented with 10% FBS. HPDE6.C7 AsPC1, Panc03.27, PSN1, and HPAFⅡ cells were grown in RPMI 1640 (Gibco) medium supplemented with 10% FBS. All the cells were incubated at 37°C and 5% CO_2_ and did not add any antibiotics in the cell culture medium.

The gemcitabine-resistant cell line of pancreatic cancer is obtained by gradually increasing the concentration of the drug treated. The BxPC3 and SW1990 cells were incubated with low concentration of gemcitabine, starting at 5 nM, and maintained until the clones did not die and continued to grow. The concentration of gemcitabine was progressively increased every 3 weeks at 10 nM. After 10 months, the final concentration of gemcitabine-containing media for BxPC3 and SW1990 reaching 200 and 50 nM, the surviving cells were considered as resistant cells and labeled as ‘BxPC3-GR’ and ‘SW1990-GR’. Correspondingly, BxPC3 and SW1990 cell lines sensitive to gemcitabine were labeled as ‘BxPC3-GS’ and ‘SW1990-GS’.

The CRISPR/Cas9 system was employed to knockout *FOXM1* gene in SW1990 cells as follows. Briefly, a DNA fragment that contained the U6 promoter, a 23-bp target sequence (5′-GTCCAATGTCAAGTAGCGGTTGG-3′) specific for *FOXM1*, a guide RNA scaffold, and a U6 termination signal sequence was synthesized and subcloned into the pGEM-T Easy vector (Promega, Madison, WI, U.S.A.). The vector was transfected with the human codon-optimized Cas9 expression vector and the enhanced GFP (eGFP) plasmid into SW1990 cells. Twenty-four hours later, GFP-positive cells were isolated by FACS and subjected to cloning by limiting dilution. After 10–14 days, knockout clones were identified after screening by Western blotting.

### Cell viability assay

Pancreatic cancer cells were seeded at a density of 5 × 10^3^ cells per well in 96-well plates. After 24 h, the cells were treated with the indicated regents. Forty-eight hours later, 10 μl of Cell Counting Kit-8 (CCK-8) reagent (Dojindo, Kumamoto, Japan) was added into each well, and the plates were cultured for another 4 h. Thereafter, the absorbance of each well was measured at 450 nm.

### Apoptosis study

The Annexin V- FITC and propidium iodide (PI) Apoptosis Detection Kits (Annexin V-FITC and PI; Dojindo) were used to monitor the apoptosis level using flow cytometry (BD Biosciences, Bedford, MA, U.S.A.). Cells were cultured with gemcitabine (100 nM) for 24 h. Cells were collected, washed with PBS, and suspended in 100 μl binding buffer, and stained with 5 μl Annexin V-FITC and PI for 15 min in the dark. The stained cells were analyzed within 1 h using FACS.

### Dual luciferase reporter assay

Pancreatic cancer cells were seeded in 24-well plates, and NF-κB activity reporter plasmids (Beyotime Biotechnology, Wuhan, China) were co-transfected with a *Renilla* luciferase expression plasmid (pRL-TK; Promega) as transfection controls. Cells were cultured with or without gemcitabine for 24 h following transfection, and the luciferase activity was measured using the Dual Luciferase Reporter Assay System (Promega). The relative promoter activity was calculated as firefly luminescence/*Renilla* luminescence.

The *FOXM1* promoter (−1000/+1 relative to the transcription start site) [18] containing a STAT1 binding site (−160/−150 relative to the transcription start site) was synthesized and ligated into pGL4.0 basic reporter vector (Promega) to create Binding site-WT. A reporter vector containing a mutated pSTAT1 binding site in the *FOXM1* promoter was constructed (Binding site-MUT: TTCCCCCACAA → GGAAAAAGTCC). Reporter plasmids were co-transfected with a *Renilla* luciferase expression plasmid (pRL-TK; Promega) as a transfection control. Cells were cultured for 24 h following transfection and treated with or without IFNγ (PeproTech, New Jersey, U.S.A.) for 6 h. Luciferase activity was measured using the Dual Luciferase Reporter Assay System (Promega). The relative promoter activity was calculated as firefly luminescence/*Renilla* luminescence.

### Quantitative real-time PCR assay

Total RNA was extracted by using the TRIzol reagent (Thermo Fisher Scientific, Waltham, MA, U.S.A.), and the reverse-transcription reactions were performed using an M-MLV Reverse Transcriptase kit (Invitrogen, Carlsbad, CA, U.S.A.). Real-time PCR was performed using a standard SYBR Green PCR kit (Toyobo Life Science, Shanghai, China). Quantitative real-time PCR (qPCR) was performed for *FOXM1* and *ACTB* (β-actin). *ACTB* expression was used as a reference to determine fold changes for the target genes using the comparative *C*_t_ method. Primer sequence (5′–3′): *FOXM1*-F: ATACGTGGATTGAGGACCACT, *FOXM1*-R: TCCAATGTCAAGTAGCGGTTG; *ACTB* -F: CATGTACGTTGCTATCCAGGC, *ACTB* -R: CTCCTTAATGTCACGCACGAT. FOXM1 quantitative primer sequence (5′–3′) for SW1990-WT/FK cells: *FOXM1*-F: TCTATACGTGGATTGAGGACC, *FOXM1*-R: ATGTCAAGTAGCGGTTGG.

### Western blotting

Total proteins were harvested from cultured cells using an ice-cold lysis buffer. Proteins were separated by 10% SDS/PAGE and then transferred to PVDF membranes. The membranes were blocked with 5% nonfat milk, and then incubated with primary antibodies against FOXM1, STAT1, P65, p-P65 (Cell Signaling Techonology, Beverly, MA, U.S.A.), pSTAT1 (Affinity, Changzhou, China), Bcl-2, Bax, Survivin, c-Myc (Proteintech, Chicago, IL, U.S.A.), and β-actin (Santa Cruz Biotechnology, lnc., Dallas, TX, U.S.A.), followed by horseradish peroxidase (HRP)–conjugated secondary antibodies (Proteintech). Immunoreactive proteins were detected using a chemiluminescence solution (Thermo Fisher Scientific).

### Immunohistochemistry

Paraffin-embedded 4-μm-thick sections were deparaffinized, heated in citrate buffer (0.01 M), treated with 0.3% H_2_O_2_ (v/v), and re-hydrated. After blocking, the sections were incubated with anti-FOXM1 antibody (1:100 dilution, Cell Signaling Techonology) or anti-pSTAT1 antibody (1:100 dilution, Affinity) in a humid chamber at 4°C overnight. After several rinses in PBS, the sections were incubated in the biotinylated secondary antibody. Subsequently, the slides were rinsed in PBS, exposed to diaminobenzidine, and counterstained with Hematoxylin. After serial dehydration, the slides were mounted for microscopic examination. As a negative control for the staining procedure, the primary antibody was omitted. The immunohistochemistry (IHC) scoring method refers to the immunoreactive score (IRS) system [31,32]. The details are as follows: the intensity of FOXM1 staining was scored as 0 (no signal), 1 (weak), 2 (moderate), and 3 (marked). Percentage scores were assigned by the percentage of stained cells in a chosen field, as 1, 0–25%; 2, 26–50%; 3, 51–75%; and 4, 76–100%. The scores of each tumor sample were multiplied to give a final score of 0–14, and the tumors were finally determined according to their FOXM1 expression as low expression, score < 9, and positive expression, score ≥ 9. Two pathologists, without prior knowledge of the clinical data, independently graded the staining intensity in all cases.

### Chromatin immunoprecipitation

SW1990 cells (3 × 10^6^) were seeded in 100 mm dishes and cultured with 50 ng/ml IFNγ for 24 h. After 1% formaldehyde treatment, the cells were lysed by 600 μl radioimmunoprecipitation assay (RIPA) lysis buffer. Genomic DNA was isolated and sheared into 200–600 bp fragments using sonication. After centrifugation, the supernatants were taken and chromatin was incubated and precipitated with antibodies recognizing pSTAT1 (Tyr^701^) (Cell Signaling Techonology) or IgG (Beyotime Biotechnology) at 4°C overnight. The immune complexes were then precipitated using protein A/G-Sepharose beads (GE Healthcare, Chicago, U.S.A.) for 4 h. Next the immune complexes were washed with different washing buffers, followed by a low salt washing buffer, high salt washing buffer, LiCl washing buffer, and TE buffer. The immune precipitates were eluted using 500 μl of elution buffer and reversal of cross-linking at 65°C overnight. *FOXM1* promoter primers were used to amplify the binding sites for pSTAT1.

### Animal experiments

Four-week-old female nude mice (BALB/c-nude) (Vital River Laboratories, Beijing, China) were housed under controlled light conditions and were allowed to feed *ad libitum*. A total of 2 × 10^6^ SW1990-WT, SW1990-FK, or BxPC3 cells in 0.1 ml PBS were injected into the left abdominal wall of mice by 1-ml syringes and 30-gauge needles. Tumor size was measured using linear calipers every 2 days. Tumor volume (V) was calculated using the following formula: length × width^2^/2. Administration of chemotherapy began when the tumor diameter reached 3–5 mm. Every Tuesday and Saturday gemcitabine (40 mg/kg) or IFNγ (10 ng/g) was injected intraperitoneally. The mice were killed 4 weeks after tumor implantation.

B-NSG mice (Biocytogen, China) were used to establish the patient-derived xenograft (PDX) models. Tumor fragments, were cut into pieces of 2–3 mm in diameter, and inoculated subcutaneously into the right flank of NSG mice. When the tumors reached approximately 100 mm^3^, the mice were randomly allocated to treatment groups. Mice were treated with gemcitabine (40 mg/kg/week) or vehicle (PBS). Tumor volumes were recorded twice weekly according to the formula: (width)2*height/2. All animal studies were conducted in accordance with the National Institutes of Health guidelines for the Care and Use of Laboratory Animals.

The tumor xenografts were removed and fixed with 4% paraformaldehyde, paraffin embedded, and sectioned at 5 μm. Immunostaining was performed to detect the FOXM1 expression in pancreatic cancer tissues using an anti-FOXM1 antibody (1:100 dilution, Cell Signaling Techonology). Immunohistochemical detection of pSTAT1 level in tissues was performed using anti-pSTAT1 antibodies (Affinity). Proliferating cell nuclear antigen (PCNA) immunostaining were performed to determine the proliferative activity of pancreatic cancer cells using anti-PCNA antibody (1:200, Arigobio, Taipei, China). Anti-Cleaved Caspase3 antibody (1:100, Cell Signaling Techonology) was used to detect the apoptotic level of cells.

### Statistical analysis

The results are presented as the mean ± standard of measurement. Statistical significance was determined using a two-tailed Student’s *t* test or ANOVA and Tukey’s *post* test. And *P*-value <0.05 was considered statistically significant. * means *P*<0.05; ** means *P*<0.01, *** means *P*<0.001. Each experiment was performed with at least three independent experiments.

## Results

### FOXM1 expression is associated with gemcitabine resistance in pancreatic cancer

**Figure 1:**
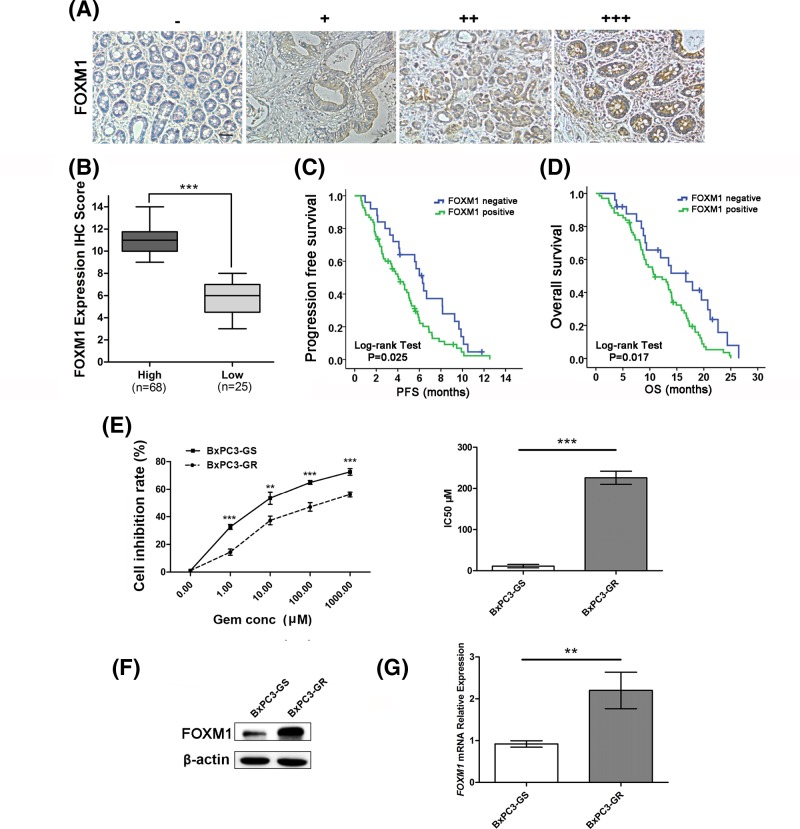
FOXM1 expression is associated with gemcitabine resistance in pancreatic cancer (**A**) Representative images of FOXM1 protein expression in paraffin-embedded tissue from 93 patients with pancreatic cancer (−, +, ++, +++). (**B**) According to the immunohistochemical score, the patients were divided into a high-FOXM1 expression group (*n*=68) and a low-FOXM1 expression group (*n*=25). (**C**) The PFS curves for the high-FOXM1 expression group and the low-FOXM1 expression group. The PFS rates between the two groups were significantly different (*P*=0.025). (**D**) The OS curves for the high-FOXM1 expression group and the low-FOXM1 expression group. The difference is statistically significant (*P*=0.017). (**E**) BxPC3-GS and BxPC3-GR cells were treated with different concentrations of gemcitabine for 48 h. Cell viability was determined using CCK-8 assays. (**F**) Western blotting analysis to determine the relative protein levels of FOXM1 in BxPC3-GS and BxPC3-GR cells. (**G**) Relative mRNA expression levels of *FOXM1* between BxPC3-GS and BxPC3-GR cell lines were compared by qPCR. Overexpression of *FOXM1* mRNA was confirmed in BxPC3-GR cells. Scale bar, 100 μm. **P<0.01; ***P<0.001.

First, we investigated the levels of FOXM1 in 93 human pancreatic cancer tissue samples using immunohistochemical analysis; all these patients received gemcitabine-based monotherapy (*n*=57, 61.3%) or combined chemotherapy (*n*=36, 38.7%). General clinical information for the patients is shown in additional Supplementary Table S1. Representative immunostaining images for weak and strong FOXM1 staining in pancreatic cancer tissues are shown in Figure 1A. FOXM1 staining was mainly located in the cytoplasm and the nucleus. Most pancreatic cancer patients’ samples were positive for FOXM1 expression, only 25 cases (26.9%) were low (Figure 1B). The progression-free survival (PFS) and OS curves calculated using the Kaplan–Meier method according to FOXM1 expression shown in Figure 1C,D. For the patients treated with gemcitabine chemotherapy, cases with low FOXM1 staining exhibited significantly better survival than patients with positive FOXM1 staining (Median PFS: 6.30 compared with 4.07 months, Log-rank test *P*=0.025; and Median OS: 16.67 compared with 10.93 months, Log-rank test *P*=0.017).

To investigate whether gemcitabine resistance was related to FOXM1 expression levels, Western blotting and qPCR analysis were performed. First we established gemcitabine-resistant cell lines, BxPC3-GR and SW1990-GR. Cell growth inhibition was then evaluated using CCK-8 assays. The resistance of BxPC3-GR and SW1990-GR cells to gemcitabine were significantly higher than that of BxPC3-GS and SW1990-GS (BxPC3, IC_50_: 10.25 ± 4.17 compared with 216.3 ± 15.89 μM, *P*<0.001, Figure 1E, SW1990, IC_50_: 2.06 ± 0.21 compared with 32.93 ± 10.50 μM, *P*<0.01, Supplementary Figure S1A). FOXM1 protein levels in gemcitabine-resistant cell lines were markedly higher than those in sensitive cells (Figure 1F and Supplementary Figure S1B) and the mRNA expression showed as similar trend, as confirmed by qPCR (Figure 1G and Supplementary Figure S1B).

In addition, we selected a normal pancreatic ductal epithelial cell (HPDE6.C7) and a panel of pancreatic cancer cell lines (AsPC1, BxPC3, Capan1, Capan2, CFPAC1, HPAF II, MIAPaca2, Panc1, Panc03.27, PSN1, PL45, SW1990, Hs766T) to detect the mRNA level of FOXM1 and the IC_50_ for gemcitabine, and analyzed the correlation between the two. It was found that all pancreatic cancer cell lines exhibited higher levels of FOXM1 expression and resistance to gemcitabine than normal pancreatic ductal epithelial cells. Moreover, in most cell lines, as the expression of FOXM1 increased, the resistance of this cell line to gemcitabine was also elevated and it can be confirmed that there is a positive correlation between the two (Supplementary Figure S1C,D).

These results indicated that FOXM1 expression might be associated with gemcitabine resistance in pancreatic cancer.

### FOXM1 decreased the sensitivity of pancreatic cancer cells to gemcitabine *in vitro* and *in vivo*

**Figure 2:**
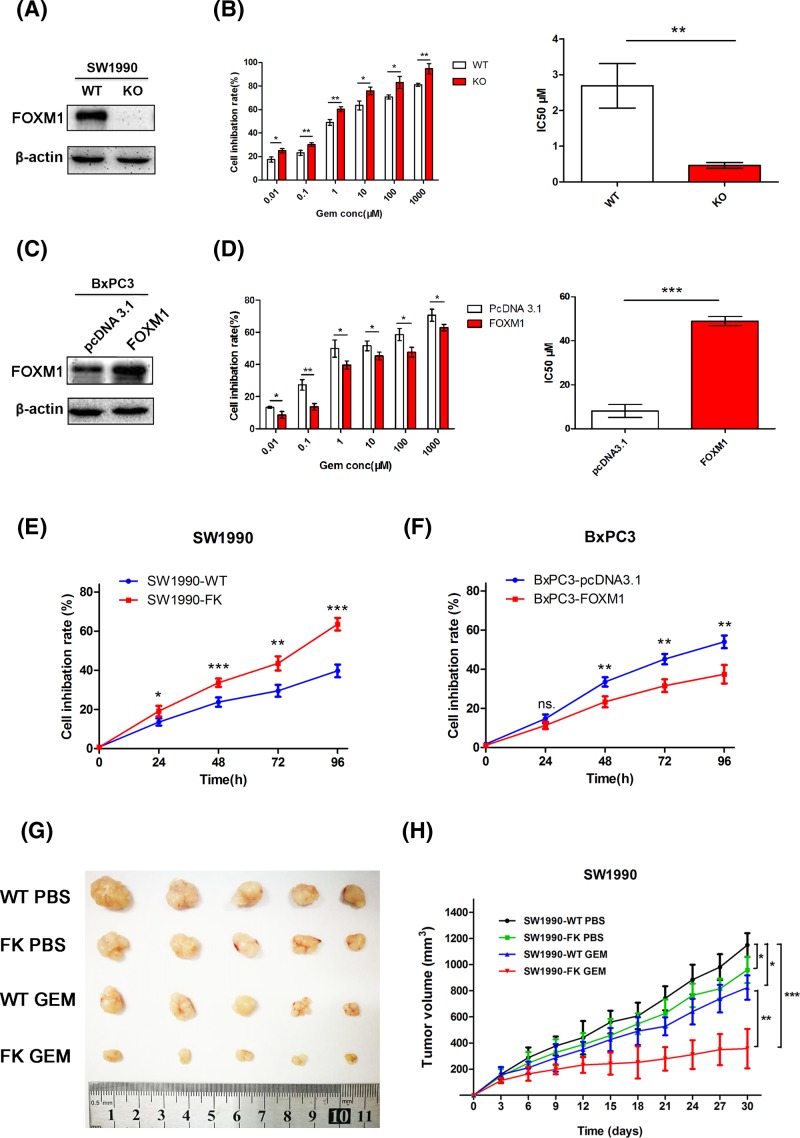
FOXM1 decreased the sensitivity of pancreatic cancer cells to gemcitabine *in vitro* and *in vivo* (**A,C**) Western blotting analysis to determine the relative protein levels of FOXM1 in SW1990-WT/FK, BxPC3-pcDNA3.1/FOXM1 cells. (**B,D**) CCK-8 assays were performed to detect the viability these cells treated with varying concentrations of gemcitabine for 48 h and to calculate the IC_50_. (**E,F**) SW1990-WT/FK and BxPC3-pcDNA3.1/FOXM1 cell lines were treated with DMSO or 100 nM gemcitabine for 4 days and cell inhibition rate at each time point was measured with CCK-8. (**G,H**) SW1990-WT/FK cells were subcutaneously injected into the left flank of nude mice. Administration of chemotherapy began when the tumor diameter reached 3–5 mm. The mice were randomly divided into four groups (*n*=5) and treated as described in figure. (**G**) Tumor size was shown after approximately 30 days of treatment. (**H**) Tumor volumes were measured every 3 days. A tumor growth curve was drawn according the measured tumor volume. *P<0.05; **P<0.01; ***P<0.001.

To further assess the effect of FOXM1 on the sensitivity of pancreatic cancer cells to chemotherapy, we generated human pancreatic cancer cells that overexpressed FOXM1 (BxPC3-FOXM1) and cells in which FOXM1 had been knocked out by CRISPR/Cas9 (SW1990-KO). Immunoblotting confirmed that the expression of FOXM1 in BxPC3-FOXM1 cells exceeded that of parental BxPC3-pcDNA3.1 cells and that SW1990-KO cells did not express FOXM1 (Figure 2A,C). To determine the effect of these changes in FOXM1 expression on gemcitabine sensitivity, CCK-8 assays were performed to detect cell viability. Both SW1990-WT/KO cells and BxPC3-pcDNA3.1/FOXM1 cells were exposed to various concentrations of gemcitabine for 48 h. The results showed that FOXM1 inhibition resulted in an obvious decrease in cell viability (IC_50_: 2.69 ± 0.62 compared with 0.46 ± 0.08 μM, *P*<0.001, Figure 2B); however, overexpression of FOXM1 increased gemcitabine resistance in BxPC3 cells (IC_50_: 8.12 ± 2.92 compared with 48.90 ± 2.07 μM, *P*<0.001, Figure 2D). To determine whether the effect of altered FOXM1 expression on gemcitabine sensitivity is time dependent, these two recombinant cell lines shown in Figure 2A,C, were subsequently used for chemosensitivity assays. The results showed, after 24, 48, 72, and 96 h of treatment by gemcitabine, SW1990 cells’ growth was more greatly inhibited by gemcitabine after FOXM1 was knocked out (Figure 2E). Induced FOXM1 expression was shown to significantly reduce the sensitivity of BxPC3 cells to gemcitabine after 48, 72, and 96 h of treatment (Figure 2F). These findings indicate that FOXM1 plays an important role in gemcitabine resistance *in vitro*.

To analyze knockout FOXM1 in regulation of proliferation inhibition and gemcitabine induced cell growth inhibition *in vivo*, we inoculated SW1990-WT/FK cells into nude mice and determined the tumor size at indicated time point post-inoculation. As shown in Figure 2G,H, FOXM1 could inhibit tumor growth. However knockout cells were more sensitive to gemcitabine treatment *in vivo*. These results show that FOXM1 can not only affect the growth of pancreatic cancer cells, but also increase the resistance of pancreatic cancer cells to gemcitabine treatment.

### Pancreatic cancer tissues with high expression of FOXM1 are less sensitive to gemcitabine in PDX tumors

**Figure 3:**
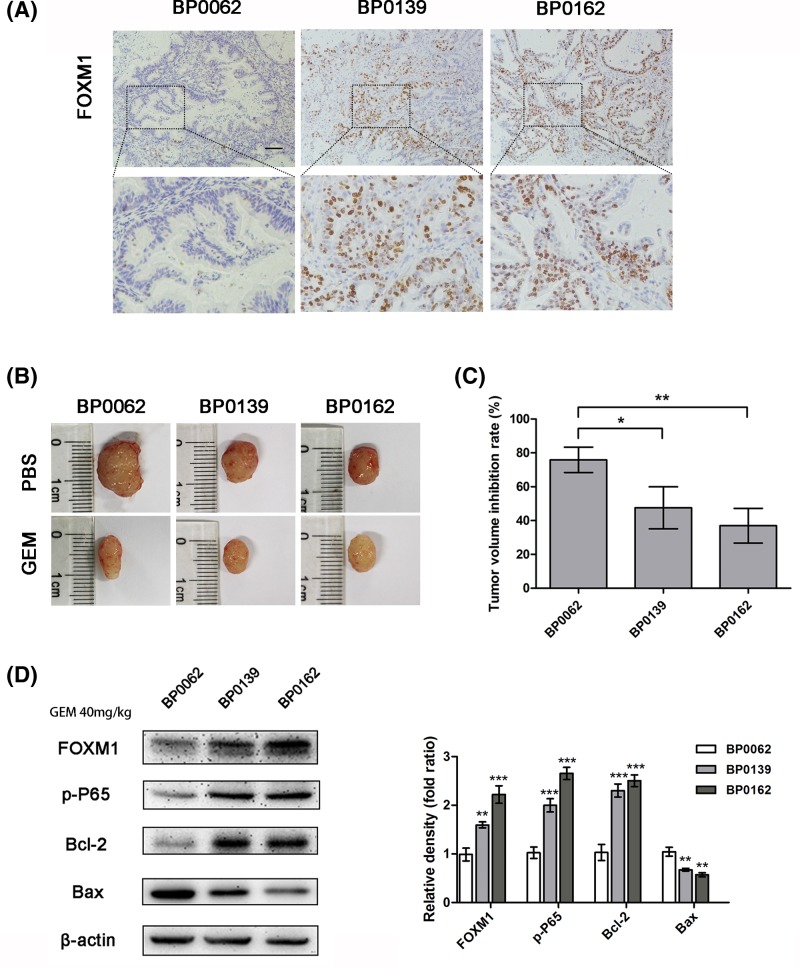
Pancreatic cancer tissues with high expression of FOXM1 are less sensitive to gemcitabine in PDX tumors (**A**) In three pancreatic cancer PDX models, FOXM1 expression levels were analyzed by IHC. Scale bar, 100 μm. (**B**) These PDX tumor sizes were shown after grouping and treatment for 3 weeks. (**C**) The inhibition rate of gemcitabine treatment for each PDX model. (**D**) The expression of FOXM1, p-P65, and apoptotic proteins Bcl2 and Bax in the tumor tissues after treatment was detected by Western blotting. *P<0.05; **P<0.01; ***P<0.001.

After establishing the pancreatic cancer PDX models successfully as described in ‘Materials and methods’ section, three PDX models with different FOXM1 level were selected to carry out experiments (Figure 3A). The results of IHC and Western blotting showed that FOXM1 expression in BP0162 and BP0139 tissues was higher than BP0062 (Figure 3A,D). Because of the heterogeneity between tumors and the different growth rates, we compared the gemcitabine susceptibility between these models by tumor inhibition rates. The results showed that, the tumors of BP0062 model (inhibition rate 75.90 ± 7.50%) were more sensitive to gemcitabine after 3 weeks of treatment with the same dose of gemcitabine, while BP0139 (inhibition rate 47.54 ± 12.41%) and BP0162 (inhibition rate 36.96 ± 10.27%) had poor response to gemcitabine (Figure 3B,C). This result suggests that in pancreatic cancer patient-derived tumor models, higher FOXM1 expression means more resistance to gemcitabine.

### FOXM1 reduced apoptosis induced by gemcitabine

**Figure 4:**
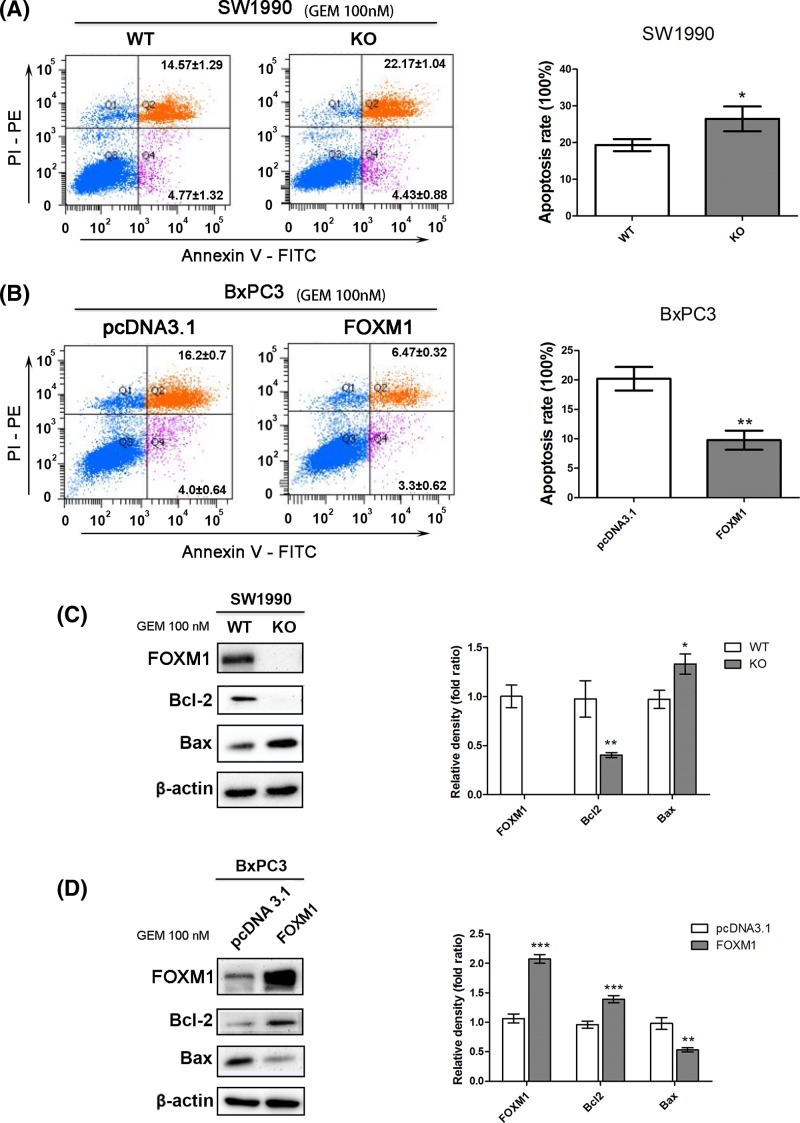
FOXM1 reduced apoptosis induced by gemcitabine (**A,B**) After treatment with 100 nM gemcitabine for 24 h, the apoptosis rates of SW1990-WT, SW1990-FK, BxPC3-pcDNA3.1, and BxPC3-FOXM1 cells were calculated using flow cytometric determination of Annexin V and PI staining. Upper-right + lower-right quadrants indicate apoptotic cells. Apoptosis is quantitated in the bar graphs for SW1990-WT and SW1990-FK (top) and BxPC3-pcDNA3.1 and BxPC3-FOXM1 (bottom). (**C,D**) Changes in the levels of apoptosis proteins, including Bcl-2 and Bax, were tested by Western blotting after FOXM1 knockout or overexpression combined with gemcitabine treatment. *P<0.05; **P<0.01; ***P<0.001.

The growth inhibition of gemcitabine on pancreatic cancer is mostly achieved by increasing apoptosis. To determine the apoptotic changes in these different FOXM1 expression PDAC cells, cellular apoptosis was analyzed in these models (the data for FOXM1 expression are shown in Supplementary Figure S2A–D) after treatment with 100 nM gemcitabine for 24 h. FOXM1 knockout significantly increased the cells’ sensitivity to gemcitabine: SW1990-KO cells exhibited significantly higher rates of apoptosis than SW1990-WT cells after treatment with 100 nM gemcitabine (26.47 ± 3.38 compared with 19.33 ± 1.67%, respectively; *P*<0.05; Figure 4A). FOXM1 overexpression significantly reduced the cells’ sensitivity to gemcitabine: BxPC3-FOXM1 cells exhibited significantly decreased apoptosis compared with that shown by BxPC3-pcDNA3.1 cells after treatment with 100 nM gemcitabine (9.77 ± 1.63 compared with 20.20 ± 2.01%, respectively; *P*<0.001; Figure 4B). However, when gemcitabine therapy was absent, FOXM1 knockdown or overexpression did not show significant difference in apoptotic rate (Supplementary Figure S2E,F).

The levels of apoptotic proteins Bcl-2 and Bax were detected by Western blotting after FOXM1 knockout or overexpression and treatment with 100 nM gemcitabine for 24 h. We found that FOXM1 overexpression could inhibit the expression of Bax and increase Bcl-2 expression to resist apoptosis (Figure 4C,D) under gemcitabine treatment. These results indicated that FOXM1 decreased the gemcitabine sensitivity of pancreatic cancer cells by inhibiting apoptosis *in vitro*.

### FOXM1 enhanced the activity of NF-κB in pancreatic cancer cells treated with gemcitabine

**Figure 5:**
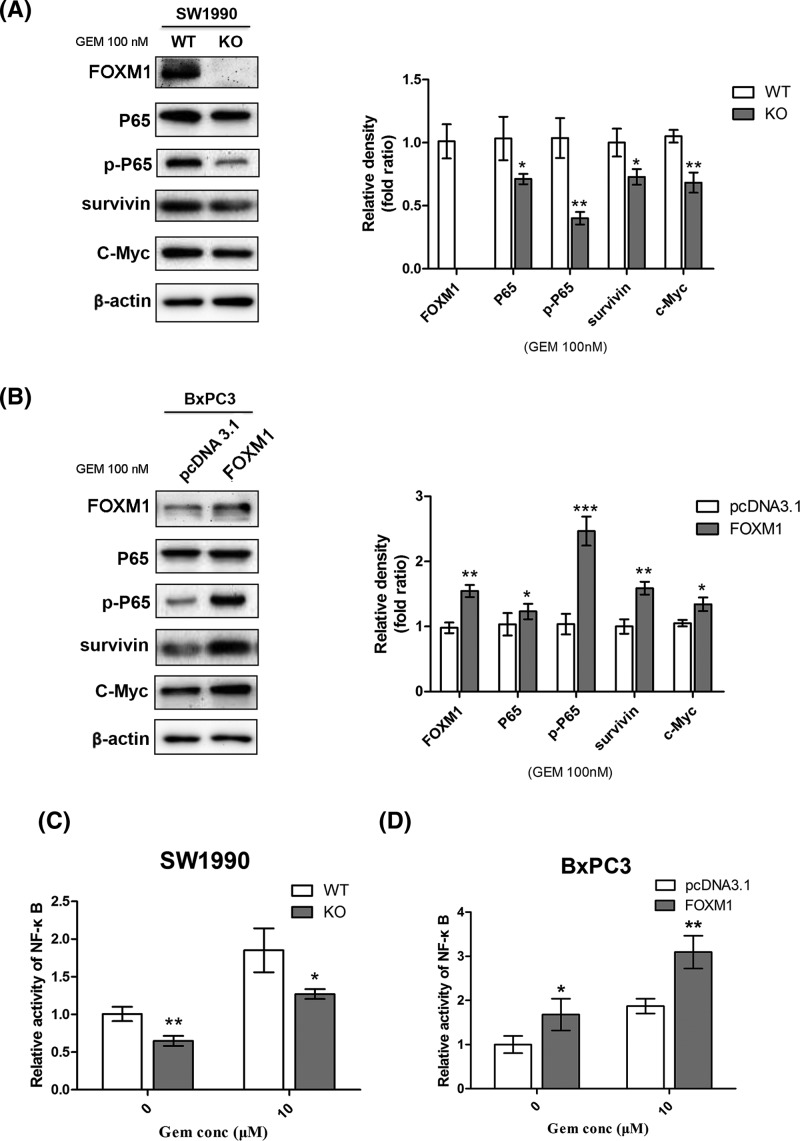
Changing FOXM1 expression influences the activity of NF-κB in cells treated with gemcitabine (**A,B**) Pancreatic cancer cells overexpressing or knocked out for FOXM1 were treated with gemcitabine, and the downstream proteins of NF-κB in the pancreatic cancer cells were detected using Western blotting analysis. (**C,D**) After the indicated treatments, the activities of the p65 subunit of NF-κB were further examined using a NF-κB dual luciferase reporter assay. Results shown are representative of three independent assays. *P<0.05; **P<0.01; ***P<0.001.

Previous studies showed that the activation of NF-κB could help pancreatic cancer cells resist chemotherapy by inhibiting apoptosis [33]. A recent study from Jin et al. [34] examined the interaction and feedback loop between NF-κB and FOXM1/β-catenin in leukemia stem cells (LSCs) in chronic myelogenous leukemia and disruption of the positive feedback loop could impair LSC self-renewal capacity and eliminate LSCs. To further determine whether the enhanced sensitivity to gemcitabine after FOXM1 inhibition correlated with changes in NF-κB activity in pancreatic cancer cells, we compared the status of NF-κB in pancreatic cancer cells using immunoblotting and NF-κB-dependent reporter activity. First, activation of NF-κB was verified by immunoblotting, which detected P65, p-P65, and downstream targets of NF-κB, such as survivin and C-Myc. Western blotting analysis showed that overexpression of FOXM1 increased the levels of P65, p-P65, and its downstream proteins. By contrast, knockout of FOXM1 suppressed these targets when the cells were treated with gemcitabine (Figure 5A,B). We further detected the effect of FOXM1 activation on NF-κB-dependent reporter activity using the dual luciferase reporter assay. The results also showed that FOXM1 inhibition suppressed the activation of NF-κB when treated with gemcitabine (Figure 5C,D). Using the PDX model, after treatment with gemcitabine, the Western blotting result showed that, tumor tissue with higher expression of FOXM1 presents higher p-P65 and Bcl2 levels and and low levels of Bax expression (Figure 3D). Taken together, our results suggested that FOXM1 overexpression could increase the activation of the NF-κB signaling cascade in pancreatic cancer cells treated with gemcitabine.

### STAT1 directly suppressed FOXM1 expression in pancreatic cancer cells

**Figure 6:**
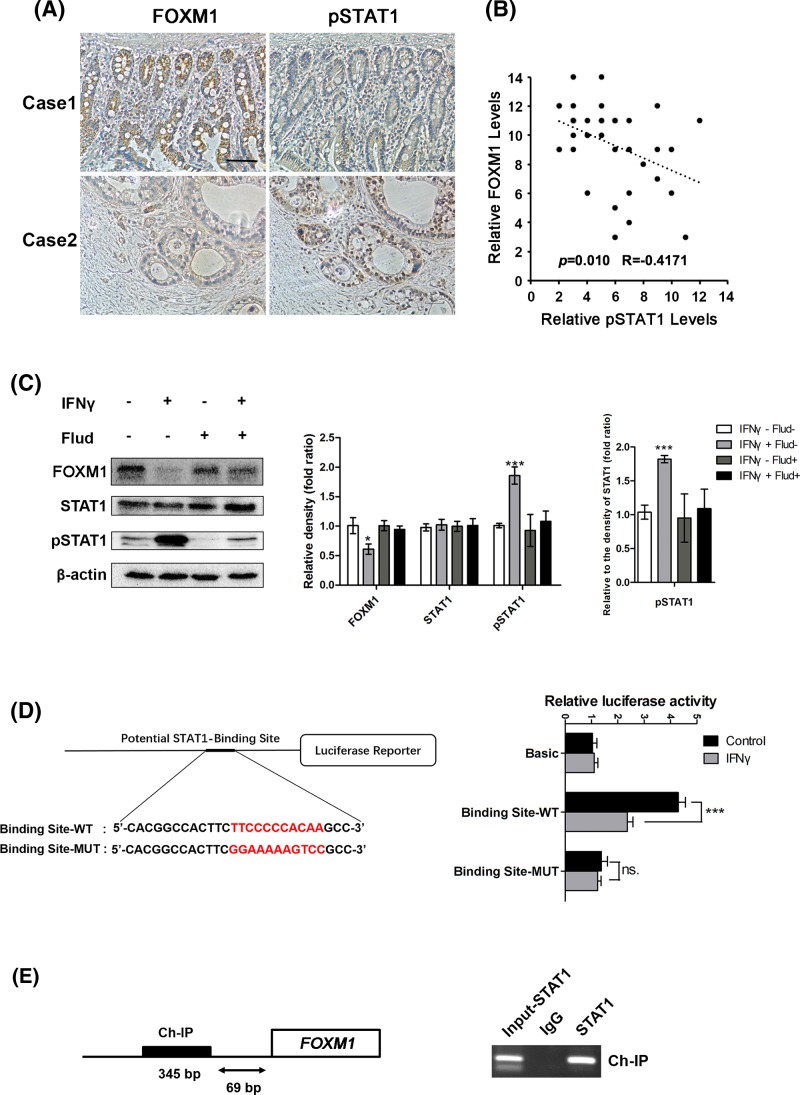
STAT1 directly suppresses FOXM1 expression in pancreatic cancer cells (**A**) Staining of the same cohorts of pancreatic tumor sections for FOXM1 expression and pSTAT1 level. (**B**) The negative correlation of FOXM1 expression with the pSTAT1 level, as assessed using Pearson correlation coefficient analysis (*n*=37, r = −0.417, *P*<0.05). (**C**) SW1990 cells were treated with IFNγ (100 ng/ml) and/or Fludarabine (10 μM) for 24 h and then whole cell lysates were extracted. FOXM1, STAT1, and pSTAT1 levels were analyzed using Western blotting. The protein levels in each well were determined quantitatively by densitometry analysis (right panels). The experiments were performed three times. (**D**) Schematic diagram of the *FOXM1* promoter luciferase reporter genes. WT, wild-type *FOXM1*-Luc reporter; MUT, mutant *FOXM1*-Luc with pSTAT1-binding sites mutated. Luciferase assays for either the wild-type *FOXM1* promoter (Binding Site-WT) or mutant *FOXM1* promoter (binding site-MUT) with or without IFNγ in SW1990 cells. Basic, empty vector control. NS, no significant difference. (**E**) 1000 bp sequence from the *FOXM1* promoter from start of transcription (+1), indicating the STAT1 bindings sites (bold boxes). Ch-IP assay demonstrating the direct binding of pSTAT1 to the FOXM1 promoter in SW1990 cells. Abbreviation: Ch-IP, chromatin immunoprecipitation. *P<0.05; ***P<0.001.

Combined with previous studies and the results above, targetting FOXM1 could promote the sensitivity of tumor cells to chemotherapy. We investigated previous studies of transcriptional regulation of FOXM1 and found that STAT3 was an important activator of FOXM1 [24]. STAT3 is a key immunomodulatory and anti-infectious transcription factor that acts downstream of interferon signaling and its effects were often opposite to those of STAT1 [30]. To investigate the relationship between phosphorylated STAT1 (pSTAT1) and FOXM1, the levels of pSTAT1 and FOXM1 were determined using IHC in pancreatic cancer tissues (Figure 6A). The 37 cases of pancreatic cancer were then divided into groups with high or low levels of pSTAT1 and FOXM1. As shown in Figure 6B, FOXM1 expression was negatively correlated with pSTAT1 level (R = −0.4171, *P*<0.05; Figure 6B). To further elucidate the connection between pSTAT1 and FOXM1 activation, SW1990 cells were treated with IFNγ or IFNα. Since phosphorylation of STAT1 is generally dependent on IFNγ or IFNα in cells, it was found in the present study that only IFNγ can inhibit FOXM1 expression (Supplementary Figure S3A). Therefore, we used IFNγ for subsequent studies. Interestingly, FOXM1 expression was suppressed, and this inhibition could be rescued by Fludarabine, which inhibits the phosphorylation of STAT1 (Figure 6C), strongly suggesting that STAT1 regulates FOXM1 expression through phosphorylation of STAT1. Moreover, FOXM1 can still be down-regulated when treated with gemcitabine and IFNγ. Fludarabine can still block this effect. However, only gemcitabine had no effect on the expression of FOXM1 (Supplementary Figure S3B).

Next, we tested whether pSTAT1 targetted *FOXM1* directly. DNA sequence analysis of 1000 bp of the *FOXM1* promoter revealed a potential STAT1 binding site. The binding site was located at nucleotides −150 to −160 bp (TTCCCCCACAA) upstream of the transcription start site. To further determine the requirement of STAT1 sites for *FOXM1* promoter activity, we explored the effect of IFNγ on *FOXM1* promoter luciferase reporters carrying the wild-type or mutant STAT1-binding sites. The mutant *FOXM1* promoter failed to elicit a response to IFNγ (Figure 6D). Chromatin immunoprecipitation (Ch-IP) assays further confirmed that pSTAT1 bound to this site in the promoter of *FOXM1* in SW1990 cells treated with IFNγ (Figure 6E). Taken together, these results indicated that the IFNγ/STAT1 pathway suppressed *FOXM1* transcription directly in pancreatic cancer cells.

### IFNγ could facilitate gemcitabine-induced cell apoptosis

**Figure 7:**
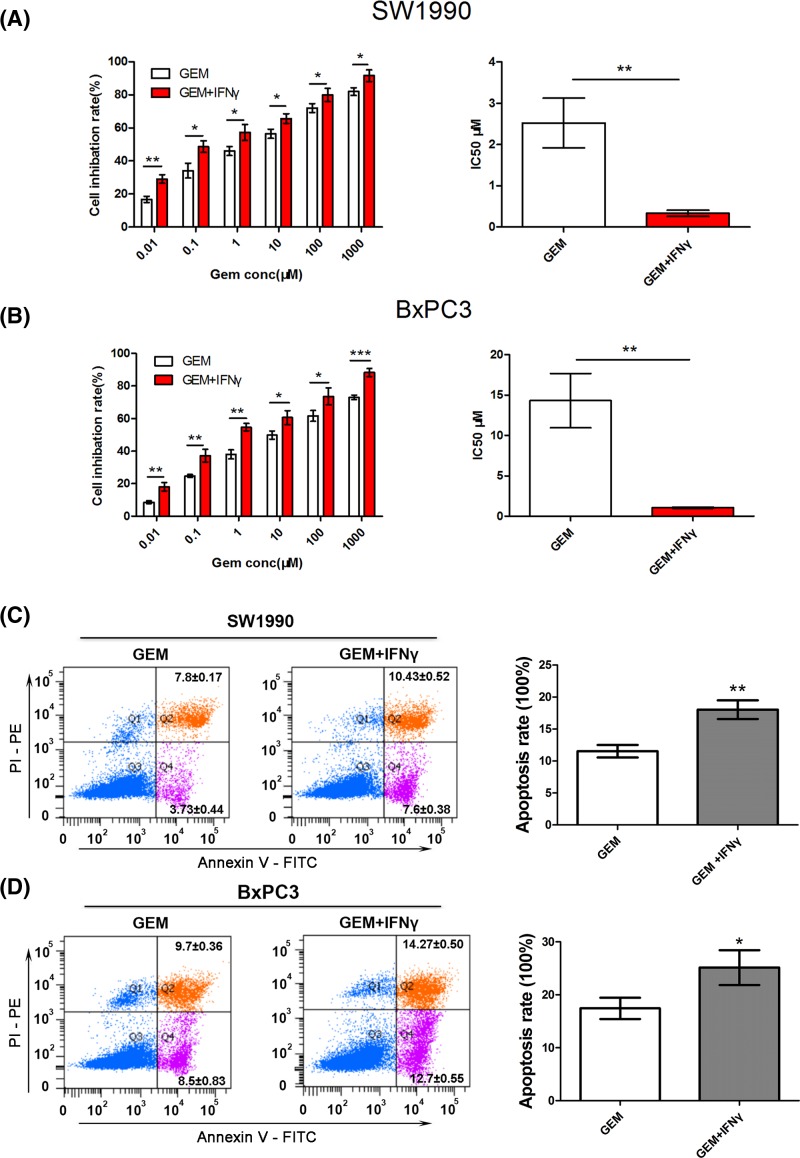
IFNγ can facilitate gemcitabine-induced cell apoptosis (**A,B**) CCK-8 assays were performed to detect SW1990 and BxPC3 cell viability after treatment with varying concentrations of gemcitabine ± 100 ng/ml IFNγ for 48 h and to calculate the IC_50_. (**C,D**) After treatment with 100 nM gemcitabine ± 100 ng/ml IFNγ for 24 h, the apoptosis rate of SW1990 and BxPC3 cells was calculated using flow cytometric determination of Annexin V and PI staining. Upper-right + lower-right quadrants indicate apoptotic cells. Apoptosis is quantitated in the bar graphs for SW1990 (top) and BxPC3 (bottom). *P<0.05; **P<0.01; ***P<0.001.

To analyze the combined effects of IFNγ and gemcitabine, SW1990 and BxPC3 cells were incubated with either gemcitabine, or gemcitabine + IFNγ, or their combination and the cell viability was detected using CCK-8 assays. Both SW1990 cells and BxPC3 cells were plated into 96-well plates and exposed to various concentrations of gemcitabine ± IFNγ for 48 h. In the two cell lines tested, improved treatment effects were seen when cells were treated with 100 ng/ml IFNγ combined with gemcitabine compared with single gemcitabine (IC_50_: 2.53 ± 0.60 compared with 0.34 ± 0.07 μM, *P*<0.001; 14.33 ± 3.35 compared with 1.08 ± 0.08 μM, *P*<0.001; Figure 7A,B). FACS was used to detect the apoptosis of the cells exposed to gemcitabine ± IFNγ. The results showed that both in SW1990 and BxPC3 cells, gemcitabine (100 nM) combined with 100 ng/ml IFNγ could significantly increase the rates of apoptosis compared with treatment by gemcitabine (100 nM) alone (18.03 ± 1.46 compared with 11.53 ± 0.99%, *P*<0.001; 25.13 ± 3.29 compared with 17.43 ± 2.01%, *P*<0.05; Figure 7C,D).

### IFNγ inhibited FOXM1 to sensitize pancreatic xenograft tumors to gemcitabine

**Figure 8:**
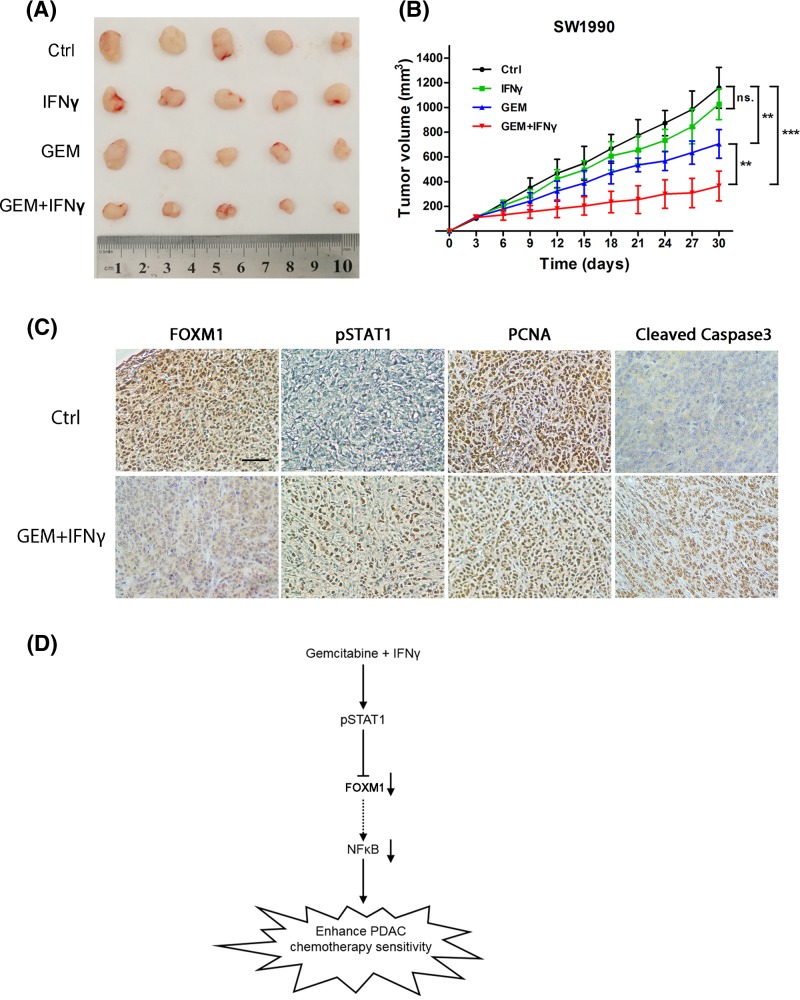
IFNγ inhibits FOXM1 sensitization to gemcitabine in pancreatic xenograft tumors (**A,B**) SW1990 cells were subcutaneously injected into the left flank of nude mice. Administration of chemotherapy began when the tumor diameter reached 3–5 mm. The mice were randomly divided into four groups (*n*=5) and treated as described in figure. (A) Tumor size was measured after approximately 30 days of treatment. (B) Tumor volumes were measured every 3 days. A tumor growth curve was drawn according the measured tumor volume. (**C**) The representative tumor tissue sections from xenografts in different treatment groups, analyzed by IHC for the expression of FOXM1, pSTAT1, the proliferation markers PCNA and apoptotic marker Cleaved Caspase3. Scale bar, 20 μm. (**D**) A schematic model showing the potential roles of IFNγ in enhancing the sensitivity of pancreatic cancer cells to gemcitabine. IFNγ suppresses the expression of FOXM1 by activating STAT1, causing inhibition of NFκB signaling, which results in increased sensitivity of pancreatic cancer cells to gemcitabine. **P<0.01; ***P<0.001.

The effect of gemcitabine treatment in combination with IFNγ on the growth of pancreatic xenograft tumors was further determined *in vivo*. SW1990 and BxPC3 pancreatic cancer xenograft models were established by subcutaneous injection of pancreatic cancer cells into the left flank of nude mice. The treatments were initiated after comparable tumor volumes were reached in the tumor-bearing mice (the tumor diameter 3–5 mm) as described in the ‘Materials and methods’ section. As shown in Figure 8A,B (Supplementary Figure S4B,C), compared with the control group, the growth rate and size of the tumors in the gemcitabine + IFNγ group were significantly reduced. However, gemcitabine alone or IFNγ alone did not significantly inhibit tumor growth *in vivo*. To further determine the efficacy of the gemcitabine + IFNγ *in vivo*, IHC was performed to analyze the FOXM1 expression and pSTAT1 level in the tumor tissue. We found decreased expression of FOXM1 after IFNγ treatment. Moreover, immunohistochemical staining of proliferation markers showed decreased PCNA staining and apoptotic marker cleaved Caspase3 showed elevated in the group treated with gemcitabine + IFNγ (Figure 8C and Supplementary Figure S4A).

## Discussion

FOXM1 is an oncogenic transcription factor and master regulator of tumor progression and metastasis [35–37]. Increasing evidence has demonstrated that FOXM1 expression often correlates with poor prognosis and chemotherapy resistance [38–41]. Studies on breast cancer showed that FOXM1 could enhance the resistance of breast cancer to chemotherapy and endocrine therapy by affecting the mitosis of tumor cells, DNA damage repair, and tumor stemness [12,13,15]. For lung cancer, Wang et al. [39] reported that FOXM1 expression could predict the outcome and sensitivity to cisplatin-based chemotherapy in patients with advanced non-small cell lung cancer (NSCLC). Tyrosine kinase inhibitors (TKIs) have demonstrated clinical benefits in the treatment of NSCLC. However, the emergence of TKI-resistance restricts their therapeutic effect. A recent study showed that TKIs could enrich cancer stem cells and induce epithelial–mesenchymal transitions, subsequently resulting in drug resistance, and these properties are associated with aberrant activation of the AKT/FOXM1/STMN1 axis [14]. Previous studies have shown that FOXM1 is essential for pancreatic cancer cells growth and survival [37,42,43]. However, there is no report of a correlation between FOXM1 and chemotherapy resistance in pancreatic cancer cells. In the present study, we demonstrated that FOXM1 expression is significantly associated with poor prognosis in patients with PDAC and with a poor response to gemcitabine-based chemotherapy. Additionally, we verified the relationship between FOXM1 and gemcitabine resistance *in vitro* and *in vivo*.

STAT3’s activity in cancer is generally viewed as oncogenic. STAT3 can promote the growth, survival, migration, or attachment of cancer cells, and might play an important role in cancer immune escape or tumor neo-vascularization [44]. In addition, STAT3 has a prominent role in mediating resistance to conventional chemo-/radiotherapies and modern targetted drugs [45]. FOXM1 and STAT3 are often related to cancer and present similar consequences when overexpressed or inhibited [46,47]. A recent study identified *FOXM1* as a new STAT3 gene target and clarified its role in proliferation, survival, drug resistance, and DNA repair in chronic myeloid leukemia [24]. Using *FOXM1* gene promoter analyses, they identified several STAT consensus-binding sequences and one STAT3-specific consensus sequence. They then demonstrated the sites as functional using EMSA, Ch-IP, and luciferase reporter assays. These results showed that FOXM1 expression is STAT3-dependent. Most of the time, STAT1 has the opposite role to STAT3. Wang et al. [30] reported that STAT3 is a key immunomodulatory and anti-infection transcription factor that acts downstream of interferon signaling, and its effects are often opposite to those of STAT1. Recently, Friedrich et al. [29] proposed a hypothesis of mutual antagonism of STAT1/STAT3, which suggested that STAT1 and STAT3 can regulate the balance of tumorigenesis. These observations suggested the FOXM1 activation might also be related to STAT1 signaling. In this study, we observed that the level of pSTAT1 was negatively correlated with the level of FOXM1 in pancreatic cancer tissue samples. Phosphorylation of STAT1 at Tyr^701^ induces STAT1 dimerization, nuclear translocation, and DNA binding [48].

In most cells, STAT1 can be activated by IFNα or IFNγ [49]. Interferons (IFNs) therapy, as an important member of tumor immunotherapy, has many years of clinical experience in tumor therapy. Clinical treatments such as melanoma [50], malignant lymphoma [51], and kidney cancer [52] have significant anti-tumor effects. Recent studies have shown that IFNs combined with other emerging therapies to treat tumors can significantly improve anti-tumor efficacy, providing a new opportunity for cancer patients. For the present study we found that IFNγ, but not IFNα, could affect FOXM1 expression (Supplementary Figure S3A). Moreover, in previous studies, IFNγ has the potential to be used clinically in the treatment of malignant glioma [53], ovarian cancer [54], and as a promising adjunct to be used to other immunotherapeutic modalities. Next, we further investigated whether IFNγ could down-regulate FOXM1 expression through STAT1 phosphorylation by targetting the *FOXM1* promoter. The results showed that *FOXM1* might be a direct gene target of STAT1.

Gemcitabine is the most widely used cross-linking drug that kills pancreatic cancer cells. Gemcitabine-based chemotherapy benefits the survival of some patients. Unfortunately, similar to other anticancer drugs, chemoresistance remains a significant drawback to its clinical success [55] and is not particularly effective in many patients with pancreatic cancer. Therefore, clarifying the mechanism of resistance to gemcitabine and exploring methods to sensitize pancreatic cancer cells to the effect of chemotherapy might enhance its efficacy and improve the prognosis for patients with pancreatic cancer. In the present study, we used immunohistochemical staining for investigating the relation between FOXM1 protein expression and resistance toward gemcitabine-based chemotherapy in patients with pancreatic cancer. We demonstrated that the FOXM1 expression is significantly associated with poor response to gemcitabine-based chemotherapy and a poor prognosis of advanced pancreatic cancer patients. These findings suggest that FOXM1 expression may involve in the molecular mechanisms of gemcitabine-based chemotherapy resistance in PDAC. However, this method has several potential limitations. First, it is a retrospective analysis from a single institution with a small sample size. This means the margin of sampling error is larger. Second, patients were treated with different chemotherapy regimens, inducing 5-Fu, DDP, OXA. But all the chemotherapy regimens were based on gemcitabine. This suggested that overexpression of FOXM1 was probably associated with gemcitabine resistance. Although we performed some experiments *in vitro* to confirm the effect of FOXM1 on the efficacy of gemcitabine alone. Further investigations are still required to clarify the effect of FoxM1 on outcome in PDAC patients. Additionally, we demonstrated that FOXM1 expression at both the mRNA and protein levels was significantly increased in BxPC3-GR (gemcitabine-resistant) cells compared with that in the parental cells. In addition, down-regulation of FOXM1 expression could enhance the sensitivity to chemotherapy *in vitro*, which indicated that knockdown of FOXM1 expression enhanced chemosensitivity of pancreatic cancer cells to gemcitabine. In addition, IFNγ could down-regulate FOXM1 through JAK/STAT1 signaling. Taken together, these results suggest that IFNγ increases the sensitivity of pancreatic cancer to gemcitabine. The *in vitro* results showed that the combination of IFNγ and gemcitabine synergistically increased apoptosis of SW1990 and BxPC3 cells. The inhibition rate was significantly higher in SW1990 or BxPC3 cells treated with IFNγ and gemcitabine than in the cells treated with gemcitabine alone. Pancreatic xenograft tumors also confirmed that the IFNγ and gemcitabine combination could significantly inhibit tumor growth in mice. Compared with gemcitabine alone, the combination group tumor growth rate was slower, and the tumor proliferation index was also significantly reduced.

In recent years, many studies have shown that FOXM1 is an essential transcription factor in promoting chemosensitivity of various solid tumors. In clinical practice, patients with pancreatic cancer, especially those with metastatic pancreatic cancer, have poor chemosensitivity. An accurate and sensitive biomarker is a better guide for doctor’s treatment. In our study, we used patients’ tissue, PDX models, and cell lines to confirm that overexpressed FOXM1 suggests pancreatic cancer resistance to gemcitabine. So, we believe that FOXM1 can be used as an effective biomarker to predict the patient’s chemosensitivity to gemcitabine and provide clues for clinicians. In addition, as an oncogene that drives pancreatic cancer development and chemotherapy resistance, FOXM1 should be more and more valued by doctors and researchers as therapeutic targets. In our research, IFNγ could be used to down-regulate the expression of FOXM1 through STAT1 phosphorylation and to increase the sensitivity to gemcitabine (Figure 8D). Therefore, FOXM1 might represent a novel target for sensitizing therapy in gemcitabine-based chemotherapy of pancreatic cancer. In addition, IFNγ is a promising agent to improve the effect of chemotherapy of pancreatic cancer.

## Clinical perspectives

- Gemcitabine is the basic chemotherapy for pancreatic cancer, but the drug resistance seriously affects the prognosis of patients. Revealing the resistance mechanism of gemcitabine and sensitizing its treatment is an urgent requirement for improving the prognosis of patients with metastatic pancreatic cancer.
- In the present study, we found that elevated expression of FOXM1 promoted the resistance of pancreatic cancer cells to gemcitabine. Fortunately, IFNγ can sensitize pancreatic cancer cells to gemcitabine via the STAT1/FOXM1/NFκB axis.
- Our results suggest that the STAT1/FOXM1/NFκB axis can inhibit chemotherapy resistance in pancreatic cancer. IFNγ is promising for sensitizing gemcitabine in patients with metastatic pancreatic cancer.

## Supporting information

**Fig S1:** 

**Fig S2:** 

**Fig S3:** 

**Fig S4:** 

**Table S1:** General information of pancreatic cancer patients (93 cases).

## Abbreviations

AKT: protein kinase B, PKB
B-NSG: NOD-Prkdcscid IL2rgtm1/Bcgen
CCK-8: cell counting kit-8
Ch-IP: chromatin immunoprecipitation
CRISPR/Cas9: Clustered Regularly Interspaced Short Palindromic Repeats / CRISPR-associated protein 9
DDP: Cisplatin
DMEM: Dulbecco’s Modified Eagle’s Medium
EMSA: Electrophoretic Mobility Shift Assay
FOXM1: forkhead box protein M1
IFNα: interferon α
IFNγ: interferon γ
IMDM: Iscove,s Modified Dulbecco,s Medium
IHC: immunohistochemistry
JAK: Janus kinase
MAPK: mitogen-activated protein kinase
MEK: mitogen-activated protein kinase kinase
NF-κB: nuclear factor κB
NSCLC: non-small cell lung cancer
OS: overall survival
OXA: Oxaliplatin
PCNA: proliferating cell nuclear antigen
PDAC: pancreatic ductal adenocarcinoma
PDX: patient-derived xenograft
PFS: progression-free survival
PI: propidium iodide
qPCR: quantitative PCR
Ras: Rat sarcoma
RPMI 1640: Roswell Park Memorial Institute 1640
STAT: signal transducer and activator of transcription
TKI: tyrosine kinase inhibitor
wnt: Wingless/Integrated
5-Fu: 5-Fluorouracil
