# Supplementary material for: STAT1-mediated inhibition of FOXM1 enhances gemcitabine sensitivity in pancreatic cancer
Source: Clin Sci (Lond). 2019 Mar 1;133(5):645–63. doi: 10.1042/CS20180816 (PMC6395369; doi:10.1042/CS20180816)
Supplement: Supplementary file 1 [file cs-133-cs20180816_supp1.pdf]

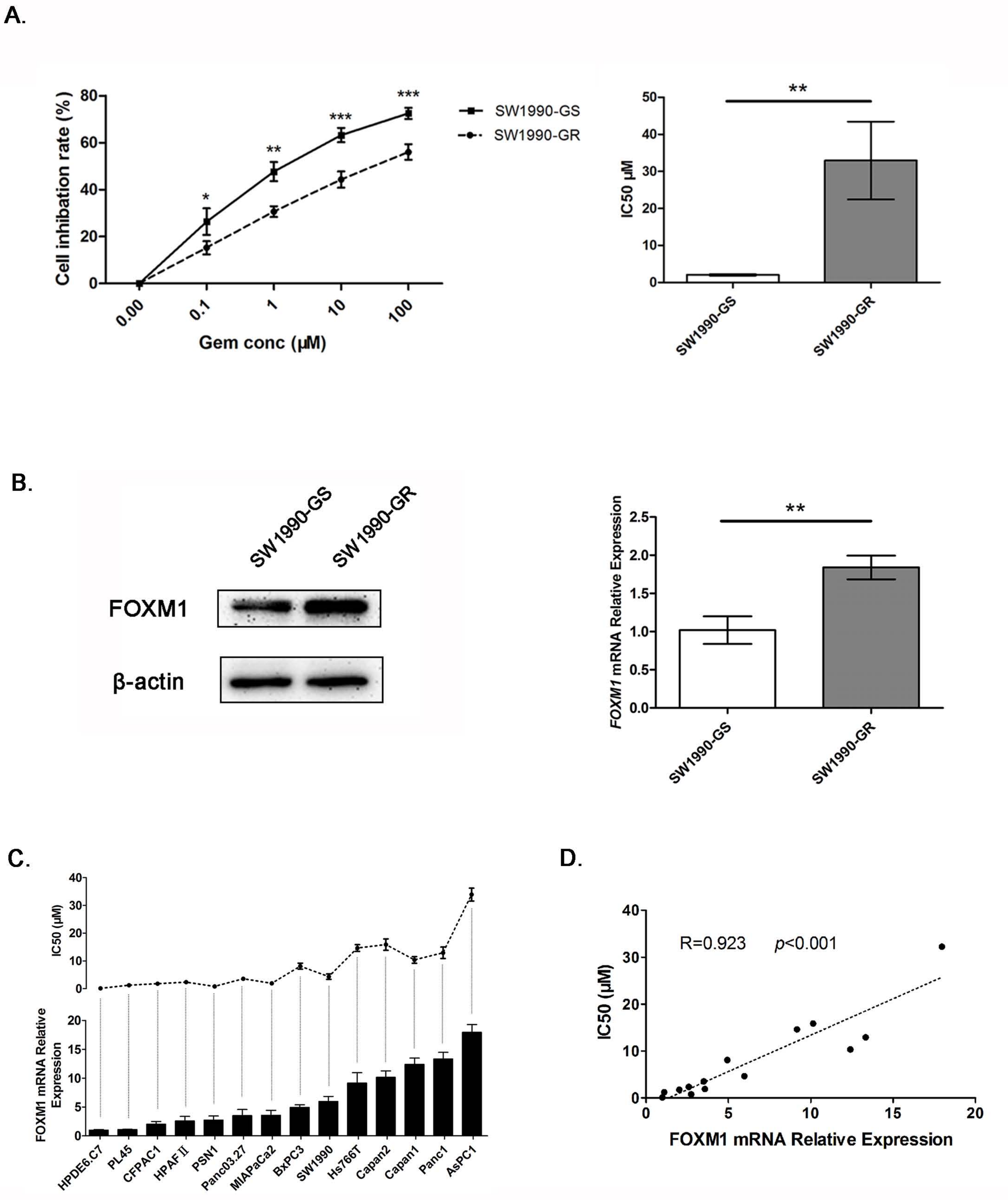
A.
Cell inhibation rate (%)
Gem conc (μM)
0.00
0.1
1
10
100
SW1990-GS
SW1990-GR
IC50 μM
B.
SW1990-GS
SW1990-GR
FOXM1
β-actin
FOXM1 mRNA Relative Expression
C.
IC50 (μM)
FOXM1 mRNA Relative Expression
HPDE6.C7
PL45
CFPAC1
HPAF Ⅱ
PSN1
Panc03.27
MIAPaCa2
BxPC3
SW1990
Hs766T
Capan2
Capan1
Panc1
AsPC1
D.
R=0.923 p<0.001
IC50 (μM)
FOXM1 mRNA Relative Expression

Fig S1

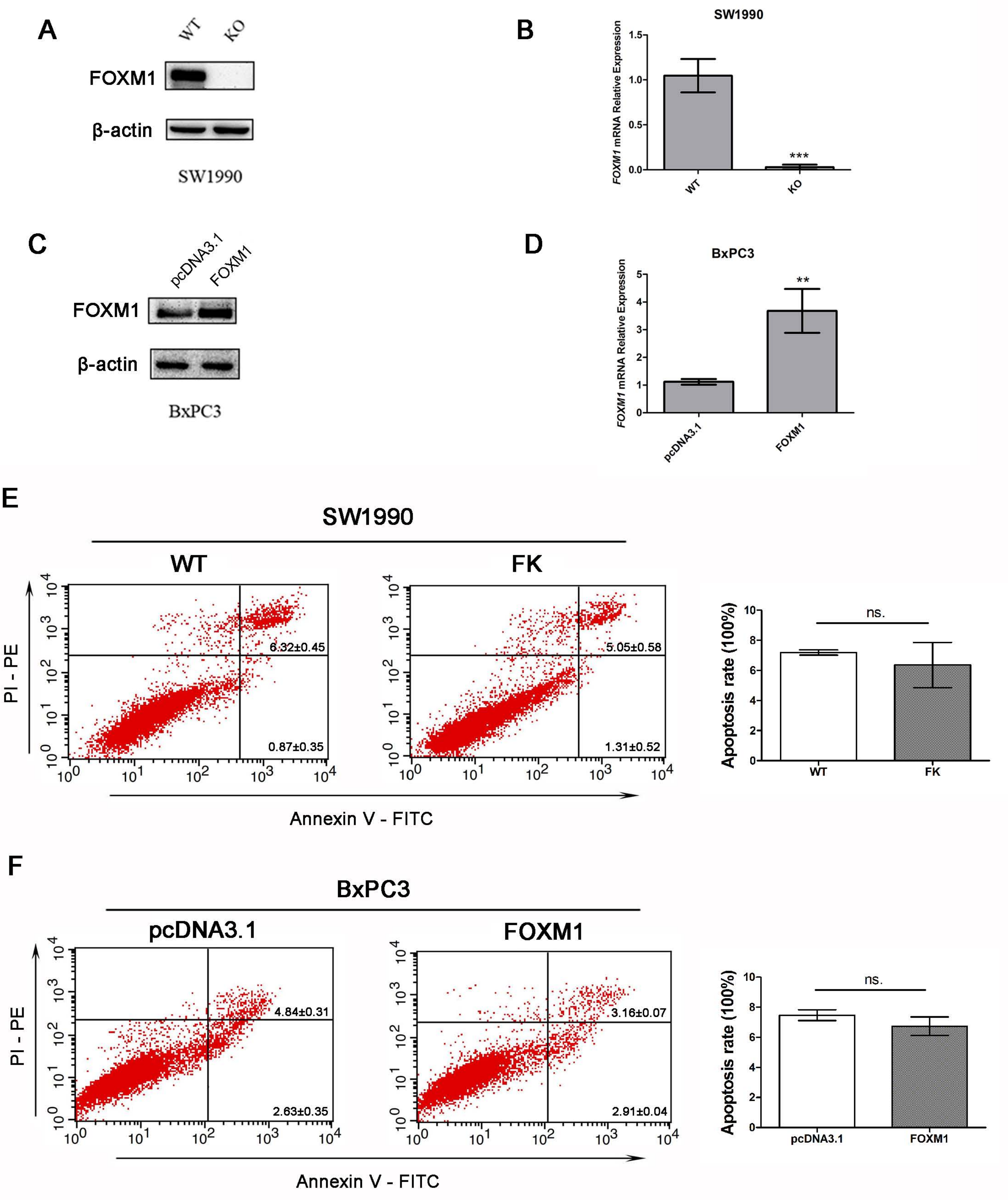
A
WT
KO
FOXM1
β-actin
SW1990
B
SW1990
FOXM1 mRNA Relative Expression
***
WT
KO
C
pcDNA3.1
FOXM1
FOXM1
β-actin
BxPC3
D
BxPC3
FOXM1 mRNA Relative Expression
**
pcDNA3.1
FOXM1
E
SW1990
WT
FK
PI - PE
6.32±0.45
0.87±0.35
5.05±0.58
1.31±0.52
Annexin V - FITC
Apoptosis rate (100%)
ns.
WT
FK
F
BxPC3
pcDNA3.1
FOXM1
PI - PE
4.84±0.31
2.63±0.35
3.16±0.07
2.91±0.04
Annexin V - FITC
Apoptosis rate (100%)
ns.
pcDNA3.1
FOXM1

Fig S2

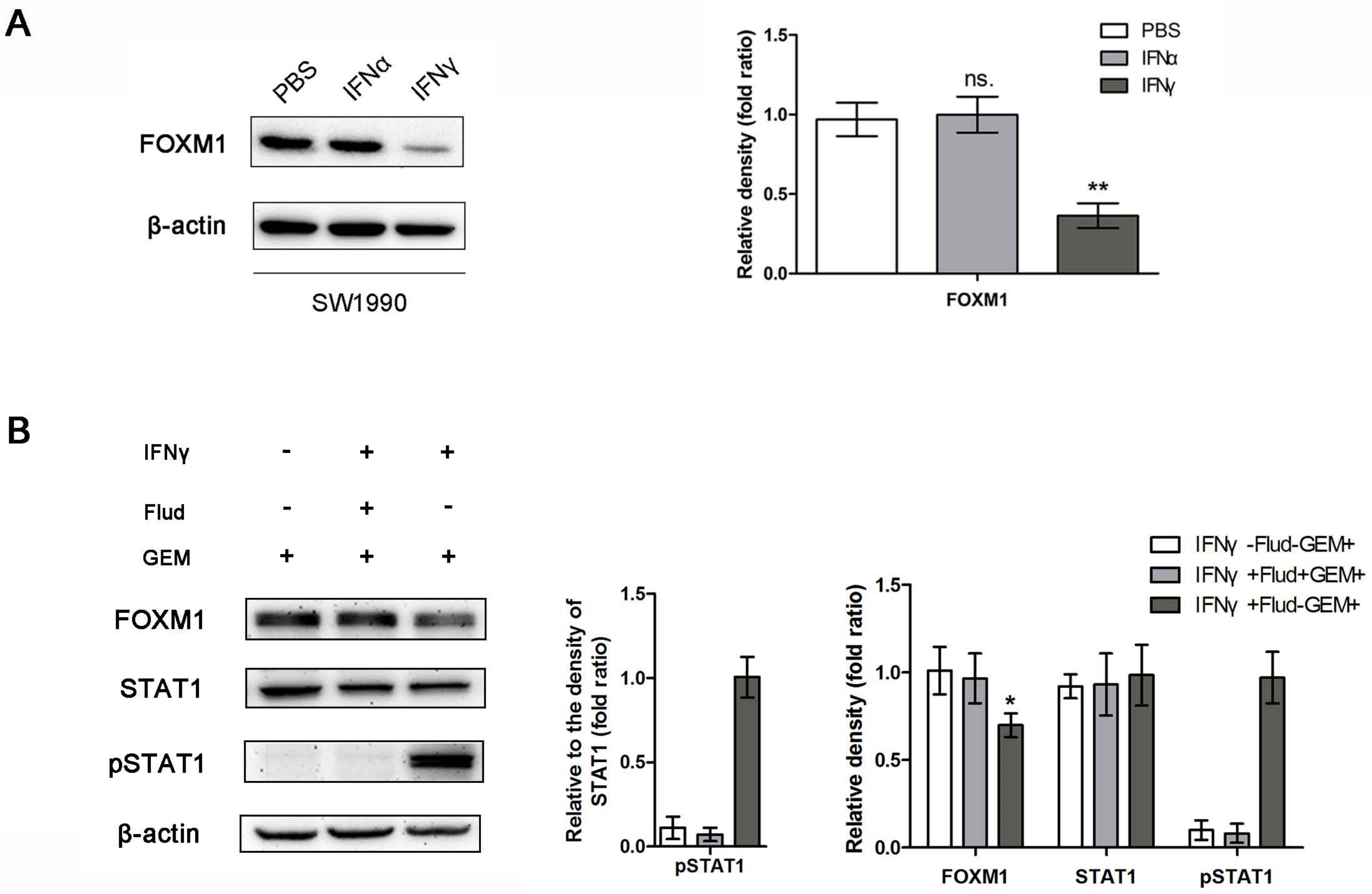
A
PBS
IFNα
IFNγ
FOXM1
β-actin
SW1990
Relative density (fold ratio)
1.5
1.0
0.5
0.0
PBS
IFNα
IFNγ
ns.
**
FOXM1
B
IFNγ - + +
Flud - + -
GEM + + +
FOXM1
STAT1
pSTAT1
β-actin
Relative to the density of STAT1 (fold ratio)
pSTAT1
Relative density (fold ratio)
IFNγ -Flud-GEM+
IFNγ +Flud+GEM+
IFNγ +Flud-GEM+
*
FOXM1
STAT1
pSTAT1

Fig S3

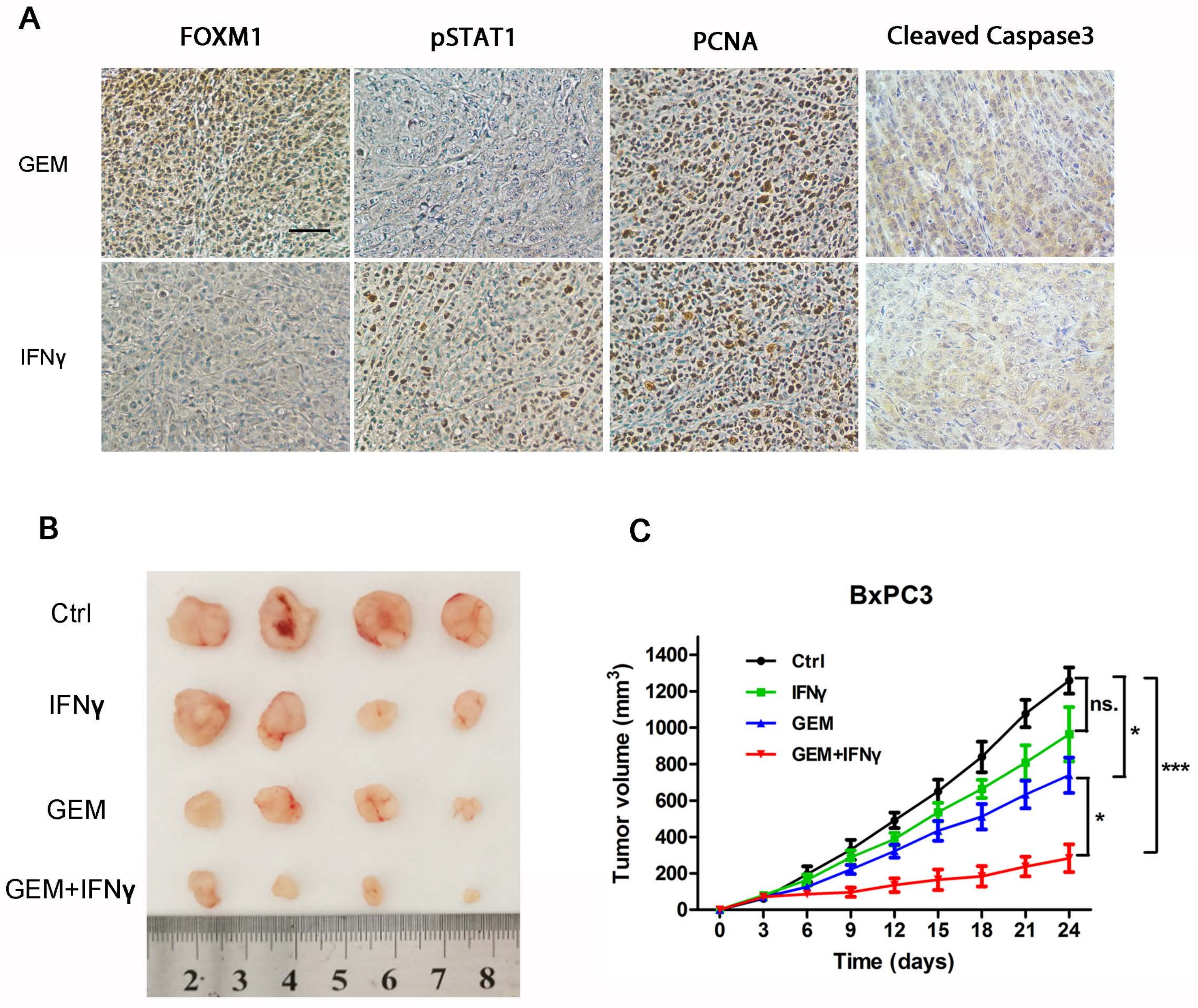
A
FOXM1
pSTAT1
PCNA
Cleaved Caspase3
GEM
IFNγ
B
Ctrl
IFNγ
GEM
GEM+IFNγ
2 3 4 5 6 7 8
C
BxPC3
Tumor volume (mm³)
Time (days)
Ctrl
IFNγ
GEM
GEM+IFNγ
ns.
*
***
*
0 200 400 600 800 1000 1200 1400
0 3 6 9 12 15 18 21 24

Fig S4

Table S1 General information of pancreatic cancer patients (93 cases).

Category	n (%)
Gender	
Male	57 (61.3)
Female	36 (38.7)
Age (years)	
>65	23 (24.7)
<65	70 (75.3)
Histological differentiation	
Well/Moderately	46 (49.5)
Poorly	47 (50.5)
Depth of invasion	
T1, 2	68 (73.1)
T3, 4	25 (26.9)
Lymph node metastasis	
Negative	76 (81.7)
Positive	17 (18.3)
Pathologic stage	
Ⅰ，Ⅱ	74 (79.6)
Ⅲ，Ⅳ	19 (20.4)
Chemotherapy regimens	
GEM	57 (61.3)
GEM+5-Fu/DDP/OXA	36 (38.7)
